# Supplementary material for: Pyruvate Dehydrogenase Kinase 1 inhibition mediated oxidative phosphorylation enhancement in cartilage promotes osteoarthritis progression
Source: BMC Musculoskelet Disord. 2023 Jul 20;24:597. doi: 10.1186/s12891-023-06585-6 (PMC10357736; doi:10.1186/s12891-023-06585-6)
Supplement: Supplementary file 1 — Additional file 1. [file 12891_2023_6585_MOESM1_ESM.zip › BP.Gsea.1653623667859/GOBP_REGULATION_OF_EPITHELIAL_CELL_APOPTOTIC_PROCESS.html]

Details for gene set GOBP\_REGULATION\_OF\_EPITHELIAL\_CELL\_APOPTOTIC\_PROCESS[GSEA]

|  || Dataset | 55457\_collapsed\_to\_symbols.cls.cls #OA\_versus\_normal.cls.cls #OA\_versus\_normal\_repos |
| Phenotype | cls.cls#OA\_versus\_normal\_repos |
| Upregulated in class | normal |
| GeneSet | GOBP\_REGULATION\_OF\_EPITHELIAL\_CELL\_APOPTOTIC\_PROCESS |
| Enrichment Score (ES) | -0.44231975 |
| Normalized Enrichment Score (NES) | -1.8553822 |
| Nominal p-value | 0.0 |
| FDR q-value | 0.065275215 |
| FWER p-Value | 0.883 |
Table: GSEA Results Summary

  

Fig 1: Enrichment plot: GOBP\_REGULATION\_OF\_EPITHELIAL\_CELL\_APOPTOTIC\_PROCESS      
 Profile of the Running ES Score & Positions of GeneSet Members on the Rank Ordered List

  

| SYMBOL | TITLE | RANK IN GENE LIST | RANK METRIC SCORE | RUNNING ES | CORE ENRICHMENT || 1 | NEUROD1 | neuronal differentiation 1 [Source:HGNC Symbol;Acc:HGNC:7762] | 1008 | 0.515 | -0.0584 | No |
| 2 | ANGPT1 | angiopoietin 1 [Source:HGNC Symbol;Acc:HGNC:484] | 1467 | 0.433 | -0.0771 | No |
| 3 | GPER1 | G protein-coupled estrogen receptor 1 [Source:HGNC Symbol;Acc:HGNC:4485] | 1677 | 0.402 | -0.0776 | No |
| 4 | ITGA4 | integrin subunit alpha 4 [Source:HGNC Symbol;Acc:HGNC:6140] | 1854 | 0.379 | -0.0765 | No |
| 5 | GAS6 | growth arrest specific 6 [Source:HGNC Symbol;Acc:HGNC:4168] | 1942 | 0.368 | -0.0688 | No |
| 6 | AKR1C3 | aldo-keto reductase family 1 member C3 [Source:HGNC Symbol;Acc:HGNC:386] | 1996 | 0.361 | -0.0587 | No |
| 7 | NFE2L2 | "nuclear factor, erythroid 2 like 2 [Source:HGNC Symbol;Acc:HGNC:7782]" | 2034 | 0.357 | -0.0476 | No |
| 8 | TNF | tumor necrosis factor [Source:HGNC Symbol;Acc:HGNC:11892] | 2098 | 0.350 | -0.0388 | No |
| 9 | SCG2 | secretogranin II [Source:HGNC Symbol;Acc:HGNC:10575] | 2156 | 0.343 | -0.0297 | No |
| 10 | NDNF | neuron derived neurotrophic factor [Source:HGNC Symbol;Acc:HGNC:26256] | 2988 | 0.251 | -0.0847 | No |
| 11 | CD160 | CD160 molecule [Source:HGNC Symbol;Acc:HGNC:17013] | 3158 | 0.234 | -0.0887 | No |
| 12 | SEMA5A | semaphorin 5A [Source:HGNC Symbol;Acc:HGNC:10736] | 3308 | 0.220 | -0.0916 | No |
| 13 | FGB | fibrinogen beta chain [Source:HGNC Symbol;Acc:HGNC:3662] | 3721 | 0.183 | -0.1166 | No |
| 14 | BAD | BCL2 associated agonist of cell death [Source:HGNC Symbol;Acc:HGNC:936] | 3788 | 0.178 | -0.1147 | No |
| 15 | FASLG | Fas ligand [Source:HGNC Symbol;Acc:HGNC:11936] | 3931 | 0.162 | -0.1194 | No |
| 16 | JAK2 | Janus kinase 2 [Source:HGNC Symbol;Acc:HGNC:6192] | 4183 | 0.140 | -0.1335 | No |
| 17 | IL11 | interleukin 11 [Source:HGNC Symbol;Acc:HGNC:5966] | 4327 | 0.126 | -0.1397 | No |
| 18 | MAPK7 | mitogen-activated protein kinase 7 [Source:HGNC Symbol;Acc:HGNC:6880] | 4396 | 0.121 | -0.1403 | No |
| 19 | TCF7L2 | transcription factor 7 like 2 [Source:HGNC Symbol;Acc:HGNC:11641] | 5034 | 0.070 | -0.1872 | No |
| 20 | YAP1 | Yes1 associated transcriptional regulator [Source:HGNC Symbol;Acc:HGNC:16262] | 5041 | 0.070 | -0.1849 | No |
| 21 | SFRP4 | secreted frizzled related protein 4 [Source:HGNC Symbol;Acc:HGNC:10778] | 5443 | 0.037 | -0.2147 | No |
| 22 | CD40LG | CD40 ligand [Source:HGNC Symbol;Acc:HGNC:11935] | 5529 | 0.030 | -0.2202 | No |
| 23 | NUPR1 | "nuclear protein 1, transcriptional regulator [Source:HGNC Symbol;Acc:HGNC:29990]" | 5616 | 0.023 | -0.2260 | No |
| 24 | TERT | telomerase reverse transcriptase [Source:HGNC Symbol;Acc:HGNC:11730] | 5773 | 0.010 | -0.2378 | No |
| 25 | MDK | midkine [Source:HGNC Symbol;Acc:HGNC:6972] | 5828 | 0.007 | -0.2417 | No |
| 26 | CAST | calpastatin [Source:HGNC Symbol;Acc:HGNC:1515] | 5941 | -0.001 | -0.2504 | No |
| 27 | PDCD4 | programmed cell death 4 [Source:HGNC Symbol;Acc:HGNC:8763] | 5961 | -0.002 | -0.2518 | No |
| 28 | FGG | fibrinogen gamma chain [Source:HGNC Symbol;Acc:HGNC:3694] | 5997 | -0.004 | -0.2544 | No |
| 29 | RB1 | RB transcriptional corepressor 1 [Source:HGNC Symbol;Acc:HGNC:9884] | 6013 | -0.006 | -0.2553 | No |
| 30 | IL4 | interleukin 4 [Source:HGNC Symbol;Acc:HGNC:6014] | 6673 | -0.054 | -0.3046 | No |
| 31 | CD40 | CD40 molecule [Source:HGNC Symbol;Acc:HGNC:11919] | 6825 | -0.065 | -0.3139 | No |
| 32 | RAMP2 | receptor activity modifying protein 2 [Source:HGNC Symbol;Acc:HGNC:9844] | 7247 | -0.099 | -0.3428 | No |
| 33 | ARRB2 | arrestin beta 2 [Source:HGNC Symbol;Acc:HGNC:712] | 7255 | -0.100 | -0.3394 | No |
| 34 | PLA2R1 | phospholipase A2 receptor 1 [Source:HGNC Symbol;Acc:HGNC:9042] | 7321 | -0.103 | -0.3404 | No |
| 35 | TNIP2 | TNFAIP3 interacting protein 2 [Source:HGNC Symbol;Acc:HGNC:19118] | 7659 | -0.131 | -0.3616 | No |
| 36 | SERPINB13 | serpin family B member 13 [Source:HGNC Symbol;Acc:HGNC:8944] | 7917 | -0.149 | -0.3758 | No |
| 37 | PLA2G1B | phospholipase A2 group IB [Source:HGNC Symbol;Acc:HGNC:9030] | 8232 | -0.173 | -0.3934 | No |
| 38 | WFS1 | wolframin ER transmembrane glycoprotein [Source:HGNC Symbol;Acc:HGNC:12762] | 8299 | -0.178 | -0.3916 | No |
| 39 | GSN | gelsolin [Source:HGNC Symbol;Acc:HGNC:4620] | 8507 | -0.194 | -0.4001 | No |
| 40 | TEK | TEK receptor tyrosine kinase [Source:HGNC Symbol;Acc:HGNC:11724] | 8623 | -0.203 | -0.4011 | No |
| 41 | CD248 | CD248 molecule [Source:HGNC Symbol;Acc:HGNC:18219] | 8816 | -0.219 | -0.4075 | No |
| 42 | IL10 | interleukin 10 [Source:HGNC Symbol;Acc:HGNC:5962] | 8855 | -0.221 | -0.4018 | No |
| 43 | FGA | fibrinogen alpha chain [Source:HGNC Symbol;Acc:HGNC:3661] | 8880 | -0.223 | -0.3949 | No |
| 44 | ABL1 | "ABL proto-oncogene 1, non-receptor tyrosine kinase [Source:HGNC Symbol;Acc:HGNC:76]" | 9081 | -0.238 | -0.4011 | No |
| 45 | EIF2S1 | eukaryotic translation initiation factor 2 subunit alpha [Source:HGNC Symbol;Acc:HGNC:3265] | 9310 | -0.256 | -0.4089 | No |
| 46 | ICAM1 | intercellular adhesion molecule 1 [Source:HGNC Symbol;Acc:HGNC:5344] | 9740 | -0.296 | -0.4307 | Yes |
| 47 | CCL2 | C-C motif chemokine ligand 2 [Source:HGNC Symbol;Acc:HGNC:10618] | 9819 | -0.303 | -0.4249 | Yes |
| 48 | CAPN10 | calpain 10 [Source:HGNC Symbol;Acc:HGNC:1477] | 9859 | -0.307 | -0.4159 | Yes |
| 49 | CDH5 | cadherin 5 [Source:HGNC Symbol;Acc:HGNC:1764] | 9946 | -0.316 | -0.4102 | Yes |
| 50 | PAK4 | p21 (RAC1) activated kinase 4 [Source:HGNC Symbol;Acc:HGNC:16059] | 10004 | -0.322 | -0.4020 | Yes |
| 51 | SERPINE1 | serpin family E member 1 [Source:HGNC Symbol;Acc:HGNC:8583] | 10284 | -0.349 | -0.4101 | Yes |
| 52 | FGF21 | fibroblast growth factor 21 [Source:HGNC Symbol;Acc:HGNC:3678] | 10341 | -0.355 | -0.4005 | Yes |
| 53 | PDX1 | pancreatic and duodenal homeobox 1 [Source:HGNC Symbol;Acc:HGNC:6107] | 10409 | -0.362 | -0.3915 | Yes |
| 54 | KRIT1 | KRIT1 ankyrin repeat containing [Source:HGNC Symbol;Acc:HGNC:1573] | 10575 | -0.381 | -0.3894 | Yes |
| 55 | SRSF6 | serine and arginine rich splicing factor 6 [Source:HGNC Symbol;Acc:HGNC:10788] | 10659 | -0.389 | -0.3806 | Yes |
| 56 | PLCG1 | phospholipase C gamma 1 [Source:HGNC Symbol;Acc:HGNC:9065] | 10722 | -0.395 | -0.3700 | Yes |
| 57 | HLA-G | "major histocompatibility complex, class I, G [Source:HGNC Symbol;Acc:HGNC:4964]" | 10869 | -0.413 | -0.3651 | Yes |
| 58 | GATA2 | GATA binding protein 2 [Source:HGNC Symbol;Acc:HGNC:4171] | 11058 | -0.439 | -0.3626 | Yes |
| 59 | PPARA | peroxisome proliferator activated receptor alpha [Source:HGNC Symbol;Acc:HGNC:9232] | 11110 | -0.445 | -0.3491 | Yes |
| 60 | FOXO3 | forkhead box O3 [Source:HGNC Symbol;Acc:HGNC:3821] | 11305 | -0.472 | -0.3457 | Yes |
| 61 | ANGPTL4 | angiopoietin like 4 [Source:HGNC Symbol;Acc:HGNC:16039] | 11389 | -0.484 | -0.3332 | Yes |
| 62 | KDR | kinase insert domain receptor [Source:HGNC Symbol;Acc:HGNC:6307] | 11454 | -0.496 | -0.3187 | Yes |
| 63 | THBS1 | thrombospondin 1 [Source:HGNC Symbol;Acc:HGNC:11785] | 11498 | -0.501 | -0.3024 | Yes |
| 64 | PRKAA1 | protein kinase AMP-activated catalytic subunit alpha 1 [Source:HGNC Symbol;Acc:HGNC:9376] | 11499 | -0.501 | -0.2827 | Yes |
| 65 | PDPK1 | 3-phosphoinositide dependent protein kinase 1 [Source:HGNC Symbol;Acc:HGNC:8816] | 11839 | -0.557 | -0.2873 | Yes |
| 66 | PIAS4 | protein inhibitor of activated STAT 4 [Source:HGNC Symbol;Acc:HGNC:17002] | 12060 | -0.610 | -0.2805 | Yes |
| 67 | CFLAR | CASP8 and FADD like apoptosis regulator [Source:HGNC Symbol;Acc:HGNC:1876] | 12079 | -0.614 | -0.2578 | Yes |
| 68 | IL13 | interleukin 13 [Source:HGNC Symbol;Acc:HGNC:5973] | 12173 | -0.636 | -0.2401 | Yes |
| 69 | GATA3 | GATA binding protein 3 [Source:HGNC Symbol;Acc:HGNC:4172] | 12276 | -0.670 | -0.2217 | Yes |
| 70 | HMOX1 | heme oxygenase 1 [Source:HGNC Symbol;Acc:HGNC:5013] | 12421 | -0.714 | -0.2049 | Yes |
| 71 | PRKCI | protein kinase C iota [Source:HGNC Symbol;Acc:HGNC:9404] | 12444 | -0.723 | -0.1783 | Yes |
| 72 | IL6 | interleukin 6 [Source:HGNC Symbol;Acc:HGNC:6018] | 12460 | -0.730 | -0.1508 | Yes |
| 73 | RGCC | regulator of cell cycle [Source:HGNC Symbol;Acc:HGNC:20369] | 12478 | -0.740 | -0.1230 | Yes |
| 74 | TNFAIP3 | TNF alpha induced protein 3 [Source:HGNC Symbol;Acc:HGNC:11896] | 12631 | -0.814 | -0.1029 | Yes |
| 75 | IGF1R | insulin like growth factor 1 receptor [Source:HGNC Symbol;Acc:HGNC:5465] | 12689 | -0.855 | -0.0738 | Yes |
| 76 | ZFP36 | ZFP36 ring finger protein [Source:HGNC Symbol;Acc:HGNC:12862] | 12846 | -1.052 | -0.0447 | Yes |
| 77 | ZFP36L1 | ZFP36 ring finger protein like 1 [Source:HGNC Symbol;Acc:HGNC:1107] | 12888 | -1.244 | 0.0009 | Yes |
Table: GSEA details [plain text format]

  

Fig 2: GOBP\_REGULATION\_OF\_EPITHELIAL\_CELL\_APOPTOTIC\_PROCESS      
 Blue-Pink O' Gram in the Space of the Analyzed GeneSet

  

Fig 3: GOBP\_REGULATION\_OF\_EPITHELIAL\_CELL\_APOPTOTIC\_PROCESS: Random ES distribution      
 Gene set null distribution of ES for **GOBP\_REGULATION\_OF\_EPITHELIAL\_CELL\_APOPTOTIC\_PROCESS**
